# Effects of 4-aminopyridine on action potentials generation in mouse sinoauricular node strips

**DOI:** 10.14814/phy2.12447

**Published:** 2015-07-08

**Authors:** Vladimir Golovko, Mikhail Gonotkov, Elena Lebedeva

**Affiliations:** Laboratory of Heart Physiology, Institute of Physiology, Komi Science Center, The Urals Branch of the Russian Academy of SciencesSyktyvkar, Russia

**Keywords:** 4-aminopyridine, mouse, sinoauricular node, transmembrane action potential, TTX

## Abstract

The physiological role of *I*_to_ has yet to be clarified. The goal of this study is to investigate the possible contribution of the transient outward current (*I*_to_) on the generation of transmembrane action potentials (APs) and the sensitivity of mouse sinoauricular node (SAN) cells to a 4-aminopyridine (4AP) as *I*_to_ blocker. The electrophysiological identification of cells was performed in the sinoauricular node artery area (*n*_strips_ = 38) of the subendocardial surface using microelectrode technique. In this study, for the first time, it was observed that dependence duration of action potential at the level of 20% repolarization (APD_20_) level under a 4AP concentration in the pacemaker SAN and auricular cells corresponds to a curve predicted by Hill’s equation. APD_20_ raised by 70% and spike duration of AP increased by 15–25%, when 4AP concentration was increased from 0.1 to 5.0 mmol/L. Auricular cells were found to be more sensitive to 4AP than true pacemaker cells. This was accompanied by a decrease in the upstroke velocity as compared to the control. Our data and previous findings in the literature lead us to hypothesize that the 4AP-sensitive current participates in the repolarization formation of pacemaker and auricular type cells. Thus, study concerning the inhibitory effects of lidocaine and TTX on APD_20_ can explain the phenomenon of the decrease in upstroke velocity, which, for the first time, was observed after exposure to 4AP. Duration of AP at the level of 20% repolarization (APD_20_) under a 4-AP concentration 0.5 mmol/L in the true pacemaker cells lengthen by 60–70% with a control.

## Introduction

The K_v_ channels are composed of four subunits each of them containing six transmembrane segments (S1–S6) and one conducting pore domain between S5 and S6 with a positively charged voltage sensor S4. The auxiliary subunit may be composed of cytoplasmic (K_v_*β* for *I*_Kur_ and *I*_to_) or single transmembrane protein (KCNE for *I*_Kr_ and *I*_Ks_) (Nerbonne and Kaas 2005). The regional differences in K^+^-channel expression contribute to the variety of morphology and duration of cardiac APs from SA area (Boyett et al. 2001) to ventricular myocytes. 4-aminopyridine (4AP) has been used in cardiac electrophysiology to study the physiological roles of I_to_ (Lei et al. 2000; Kocic 2002) and *I*_Kur_ (Yue et al. 1996). 4AP is considered selective for these currents of the heart with micromolar concentrations being used to block *I*_Kur_ (Yue et al. 1996) and millimolar concentrations to block *I*_to_ (Mitcheson and Hancox 1999). However, limited published data raise the possibility that 4AP may not be entirely selective for *I*_to_ over the rapid delayed rectifier K^+^-current (*I*_Kr_) (Mitcheson and Hancox 1999). A nonselective effect of 4AP on *I*_Kr_ would significantly influence the interpretation of data from experiments in which 4AP is applied to cardiac muscle cells. The mechanism of blocking the Kv11.1 channel by 4AP differs from the mechanism of blocking the channel current *I*_to_. Therefore, in the heart cells of the rabbit and human millimolar concentrations of 4AP may lengthen APs without regard to the effect on the current *I*_to_. It was found that 4AP in micromolar concentration had effect on the auricle cells. At the same time, the millimolar concentrations did not change the phase of final repolarization (Gonotkov et al. 2012).

The transient outward current (*I*_to_) has been identified in the sinoauricular node (SAN) of the rabbit. However, the physiological role of *I*_to_ has yet to be clarified (Uese et al. 1999; Boyett et al. 2001; Cha et al. 2009). Very little is known about the 4–aminopyridine (4AP) sensitive current in mouse sinoauricular node (Nerbonne and Kaas 2005; Gonotkov et al. 2012).

The transient outward current (*I*_to_) in mammalian ventricular cells, including those of mice, has been experimentally demonstrated to be produced by K^+^ outward ions. This current contributes largely to the formation of the early repolarization phase (phase 1) AP (Nerbonne and Kaas 2005). Phase 1 of the action potentials (APs) cannot be identified in pacemaker cells. Thus, some researchers believe that the *I*_to_ current in these cells is absent or inactivated (Verkerk and van Ginneken 2001; Cha et al. 2009). Other researchers consider that 4AP-sensitive *I*_to_ current significantly contributes to the repolarization of sarcolemma in rabbit sinoauricular node (SAN) cells (Uese et al. 1999; Lei et al. 2000).

The understanding of the physiological role of 4AP-sensitive current in pacemaker cells is important for theoretic and applied biomedicine. For example, it can be used in the development of medication for the treatment of disturbances in electrical activity by acting upon the main center of heart automaticity, that is*,* the sinoauricular node. The goal of this study is to investigate the possible contribution of the transient outward current (*I*_to_) on the generation of action potentials and the sensitivity of mouse SAN cells to a 4AP blocker.

## Methods

### Animals

Two-month-old male albino mice (*n* = 38) weighing 30 ± 5 g were purchased from the experimental animal colony of the Institute of Biology, Komi Science Centre, Syktyvkar, Russia. The study was performed in accordance with the *Guide for the Care and Use of Laboratory Animals* published by the US National Institute of Health (NIH Publication No. 85–23, revised 1996) and European directives (86/609/CEE).

### Sinoatrial area preparation

**Figure 1:**
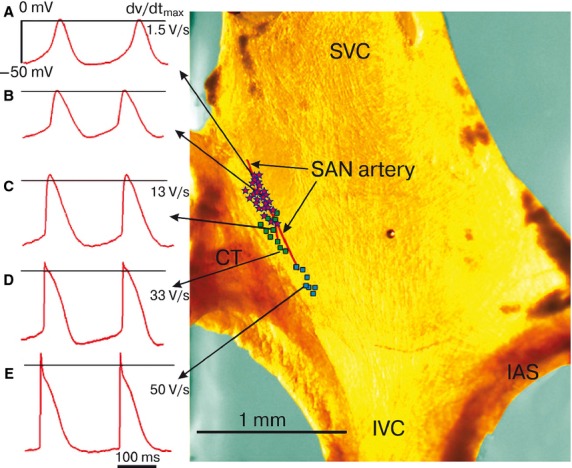
Mouse SAN area examples of intracellular APs. Photograph of a whole SAN-atrial preparation. The color of the tissue is the result of the lighting used. (A–D) examples of intracellular APs from SAN. (E) examples from right branch of the SA ring branch. The symbols used to label true pacemakers (pink asterisks), latent pacemakers (green squares) and auricle cells (blue squares). CT, crista terminalis; IAS, interatrial septum; IVC, inferior vena cava; SVC, superior vena cava.

Animals were anesthetized by ether inhalation and sacrificed by cervical dislocation. The chest was opened, and the beating heart was quickly removed followed by dissections in an oxygenated saline solution. After removing the ventricles, the right auricle was incised to expose the crista terminalis, intercaval area, and interatrial septum. The preparation was then trimmed, and both the SA area and the surrounding auricle were isolated. The final SA area preparation was approximately 3 × 2 mm (Fig.1) in size and contained the sinoatrial SAN and a small portion of the surrounding tissue of the right auricle (Gonotkov and Golovko 2011). The preparation was fixed with the subendocardial surface face-up in a 5-ml tissue bath with a perfusing Tyrode solution composed of (in mmol/L): 140 NaCl, 10 NaHCO_3_, 5.4 KCl, 1.8 CaCl_2_, 1 MgSO_4_, 10 d glucose, and 5 HEPES (adjusted to pH 7.4 with NaOH) at a rate of 8–10 ml/min. The solution was saturated with a 95% O_2_ and 5% CO_2_ gas mixture before entering a tissue bath at 31 ± 1°C.

### Electrophysiological recordings

Transmembrane APs were recorded with fixed glass microelectrodes filled with 2.5 mol/L KCl, connected through an agar-Tyrode bridge, and an AgCl wire to the input of the source follower. The initial electrode resistance ranged between 15 and 40 MΩ. The diameter of micropipette tip was controlled through a microscope (Tesla, Czech Republic). The exterior diameter did not exceed 0.2 *μ*m. Recording electrodes were fabricated from borosilicate glass.

Aminopyridine, TTX, and lidocaine (Sigma-Aldrich, Stenheim, Germany) were dissolved in deionised water. Stock solutions were stored in refrigerator at 5°C. Small aliquots of 4-aminopyridine (0.1–5 mmol/L) were inspected in the extracellular solution. Strips, which demonstrated the regular activity of a “control” solution, were employed.

The working frequency of electrical impulses registering in the analog form ranged from 0 to 5 kHz. Action potentials were recorded *via* the ADC (type E14-140, L-Card, Russia) on a hard disk drive. Action potentials with no amplitude change within 5 min of registration were considered eligible for further data processing.

### Data analysis

The following parameters were measured: maximum diastolic potential (MDP); action potential amplitude (APA); duration of action potential at the level of 20% (APD_20_), 50% (APD_50_), 90% (APD_90_), and 100% (APD_100_) repolarization; cycle length (CL); diastolic depolarization (DD); maximal upstroke velocity (d*V*/d*t*_max_); velocity of the final repolarization phase (V_3_); and diastolic depolarization rate (DDR). Data from each experiment (*n* = 8–21 cells) are expressed as the mean ± standard deviation (M ± *σ*). Statistical analyses were performed with Microsoft Office Excel and PowerGraph Professional version 3.3 (Russia) using the Wilcoxon’s paired *t*-test and Mann–Whitney *U*-test. Differences were considered significant at *P* < 0.05.

## Results

### Microelectrode identification cells of SAN artery area

The microelectrode mapping was initiated from the center, between the lateral and medial limbs, by a step of 50 *μ*m along the SA node artery (Sutyagin et al. 2005; Gonotkov and Golovko 2011). The murine SAN has a unifocal type of impulse generation and consists of heterogeneous electrical cells similar to other mammals‘ nodes (Golovko 1989; Verheijck et al. 2001; Tellez et al. 2006). The action potential configurations from the SAN artery area were ultimately categorized into one of three main types: the true, the latent pacemaker, and the atrial like AP (Table1).

**Table 1:** Action potentials electrophysiological parameters of the mouse sinoauricular area cells.

Parameters	True pacemaker cells *n* = 33	Latent pacemaker cells *n* = 25	Atrial cells *n* = 10
MDP, mV	–55 ± 5*	–62 ± 8	–79 ± 4*
APA, mV	52 ± 5*	66 ± 12	96 ± 4*
Potential threshold, mV	–42 ± 6*	–53 ± 10	–79 ± 4*
APD_20_, msec	42 ± 8*	24 ± 11	7 ± 1*
APD_50_, msec	59 ± 9*	47 ± 10	14 ± 3*
APD_90_, msec	86 ± 11*	76 ± 9	46 ± 13*
CL, msec	193 ± 17	198 ± 16	200 ± 16
DD, msec	86 ± 16	100 ± 18	−
d*V*/d*t*_max_, V/sec	3 ± 1* (1.5 ÷ 7)	27 ± 16 (10 ÷ 65)	110 ± 6* (102 ÷ 115)
V_3_, V/sec	–0.7 ± 0.2	–0.9 ± 0.2	–1.5 ± 0.3*
V_4_, mV/sec	134 ± 27*	95 ± 32	−

Abbreviations: APA, action potential amplitude; APD_20_, APD_50_, APD_90_, APD_100_, duration of action potential at the level of 20%, 50%, 90% and 100%; CL, cycle length; DD, diastolic depolarization; d*V*/d*t*_max_, maximal upstroke velocity; MDP, maximum diastolic potential; V_3_, velocity of the final repolarization phase; V_4_, diastolic depolarization rate.

Values are means ± SEM or medians.

* Significant difference compared with latent pacemaker cells.

The first type is a dominant pacemaker that possesses a maximal upstroke velocity of less than 7 V/sec, a diastolic depolarization rate of approximately, 130 mV/sec and the longest APD_90_ (Fig.1A and B; Table1). Around these dominant pacemaker myocytes, there was a zone of secondary pacemaker cells, commonly called the transitional zone. Electrophysiological features of these myocytes (type 2, Fig.1C–E) were intermediate between the dominant pacemaker cells and the auricular cells. They had a lower diastolic depolarization rate, higher upstroke velocity, higher action potential amplitude and shorter APD_90_ compared to the dominant pacemaker cells (Table1). In the transitional zone, myocytes with the auricular type of electrical activity could also be impaled. Those auricular like cells had a constant diastolic potential and did not have the early repolarization phase.

We managed to measure a few APs in cells of the right sinoauricular ring bundle. They were characterized by an early repolarization phase (phase 1), diastolic depolarization, and high upstroke velocity (Fig.1E).

### 4AP effects on AP generation

**Figure 2:**
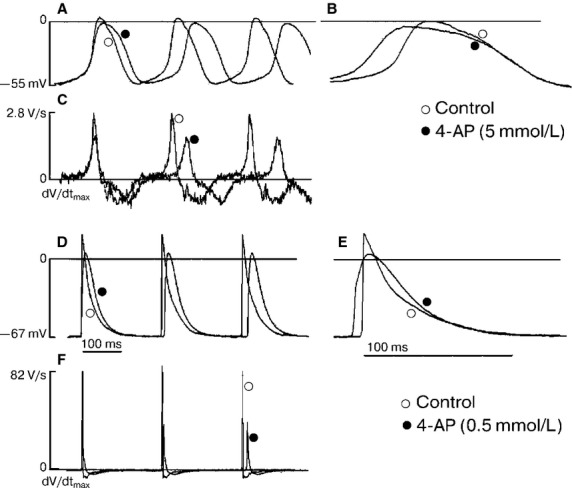
Effect of 4-AP as a specific *I*_to_ blocker. Action potentials and upstroke velocity (d*V*/d*t*_max_) of pacemaker cells (A, B, C) and auricle type cells (D, E, F) in the control and presence of 4-AP. Abbreviations: ○ – control, ● – 4-AP (0.5 and 5 mmol/L).

We thoroughly studied the dose-dependent 4AP effects in a range of 0.1–5.0 mmol/L on the main AP electrophysiological parameters of SAN artery area cells. In cells with the lowest d*V*/d*t*_max_ (3 ± 1 V∕sec), hypothetically true pacemaker 4AP (0.5 mmol/L) most visibly affected AP duration at the 20% repolarization (APD_20_) level or at the AP “plateau” phase and was prolonged from 43 ± 7 to 60 ± 10 ms (*n* = 5; *P* < 0.01). This was accompanied by decreased of the d*V*/d*t*_max_ by approximately 37% (Fig.2C and F). Thus, the AP duration was lengthened by 25%. Furthermore, the 4AP concentration increased from 1 to 5 mmol/L and caused the APD_20_ to rise by 61–73% and the d*V*/d*t*_max_ to decrease by 50%, on average (Fig.2, Table2).

**Table 2:** Effect of 4-AP on electrophysiological parameters of the true pacemaker cells.

Parameters	4-AP					
	Control *n* = 5	0.5 mmol/L *n* = 5	Control *n* = 6	1 mmol/L *n* = 6	Control *n* = 5	5 mmol/L *n* = 5
MDP, mV	−52 ± 3	−52 ± 4	−53 ± 2	−56 ± 6	−56 ± 6	−50 ± 4
APA, mV	50 ± 4	45 ± 2	48 ± 6	42 ± 4	55 ± 7	41 ± 7*
Potential threshold, mV	−44 ± 4	−45 ± 5	−45 ± 5	−48 ± 4	45 ± 4	−42 ± 4
APD_20_, msec	43 ± 7	60 ± 10*	49 ± 10	79 ± 19*	47 ± 11	76 ± 14*
APD_50_, msec	60 ± 8	79 ± 11	63 ± 9	94 ± 19*	65 ± 15	102 ± 15*
APD_90_, msec	88 ± 12	109 ± 22	87 ± 12	119 ± 17*	91 ± 18	134 ± 16*
CL, msec	194 ± 26	201 ± 43	194 ± 31	211 ± 44	194 ± 27	224 ± 9
DD, msec	85 ± 16	72 ± 23	86 ± 20	72 ± 29	79 ± 19	71 ± 21
d*V*/d*t*_max_, V/sec	2 ± 1	2 ± 1	2.3 ± 1	1.3 ± 0.3	4 ± 2	1.5 ± 0.3*
	(1.5 ÷ 3)	(1.5 ÷ 3)	(1.5 ÷ 4)	(1 ÷ 1.5)	(2.5 ÷ 6)	(1.5 ÷ 2)
V_3_, mV/sec	0.65 ± 0.3	0.65 ± 0.3	0.75 ± 0.2	0,67 ± 0,14	0.86 ± 0.3	0.44 ± 0.25
V_4_, mV/sec	134 ± 38	121 ± 54	121 ± 29	96 ± 41	144 ± 28	103 ± 16

Abbreviations: APA, action potential amplitude; APD_20_, APD_50_, APD_90_, APD_100_, duration of action potential at the level of 20%, 50%, 90% and 100%; CL, cycle length; DD, diastolic depolarization; d*V*/d*t*_max_, maximal upstroke velocity; MDP, maximum diastolic potential; V_3_, velocity of the final repolarization phase; V_4_, diastolic depolarization rate.

Values are means ± SEM or medians.

* Significant difference compared with control.

Finally, the AP duration was increased by 25%, whereas the AP generation frequency reduced by 19–23% compared to the control. Moreover, 4AP did not influence the duration of the final repolarization phase (Fig.2A and B).

**Figure 3:**
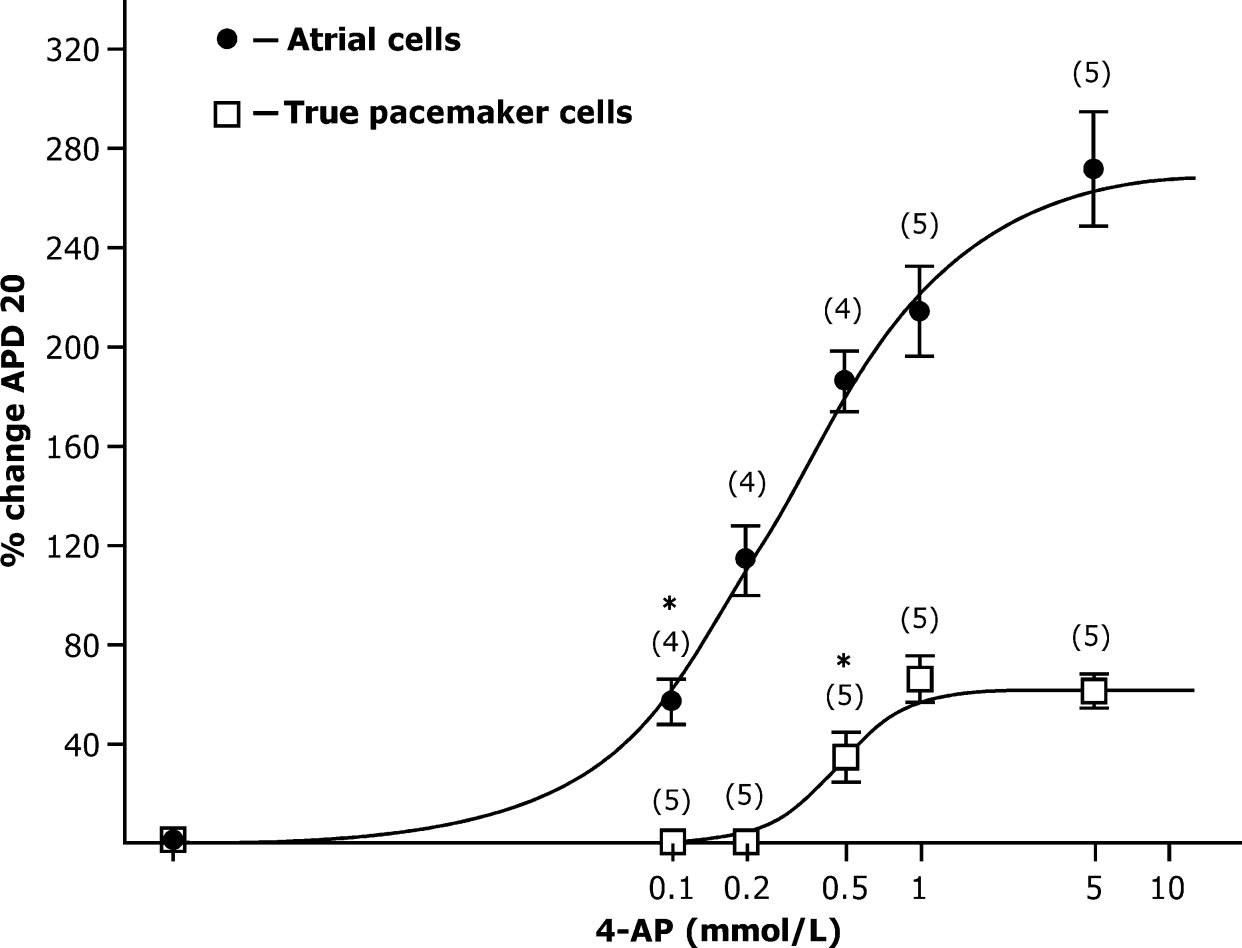
Dose-dependent effect of 4-AP (0.1–10 mmol/L) on APD_20_ in true pacemaker and auricle type cells. *P < 0.05 versus control solution, *n*, number of strips.

In a series of experiments on spontaneously beating strips, we studied the effects of 4AP on the action potential configuration of contractile myocardium auricular cells. These cells had a short period APD_20_ of 7 ± 1 msec (*n* = 10), of the AP amplitude was 96 ± 4 mV, and no diastolic depolarization. The 4AP blocker (0.1 mmol/L) was prolonged APD_20_ by 1.5 times, but d*V*/d*t*_max_ did not significantly change compared to the control (*n* = 5; *P* > 0.05; Fig.2D and E). Further increases in 4AP concentration (0.2, 0.5 and 1.0 mmol/L) caused a dose-dependent triple rise in the duration of the AP “plateau” phase (Fig.3). We identified that the APD_20_ dependence on 4AP concentration corresponded to the curve predicted by Hill‘s equation. This was accompanied by a d*V*/d*t*_max_ and AP amplitude decrease of 15–20% as compared to the control.

Consequently, the “plateau” phase AP of pacemaker cells and auricular cells lengthened by more than 50% at 4AP concentrations of 0.6 and 0.1, respectively. Thus, the shorter the initial AP “plateau” phase duration in the cell was the higher cell’s sensitivity to transient outward K^+^–current (*I*_to_) inhibitors.

### Lidocaine and TTX effects on AP generation

**Figure 4:**
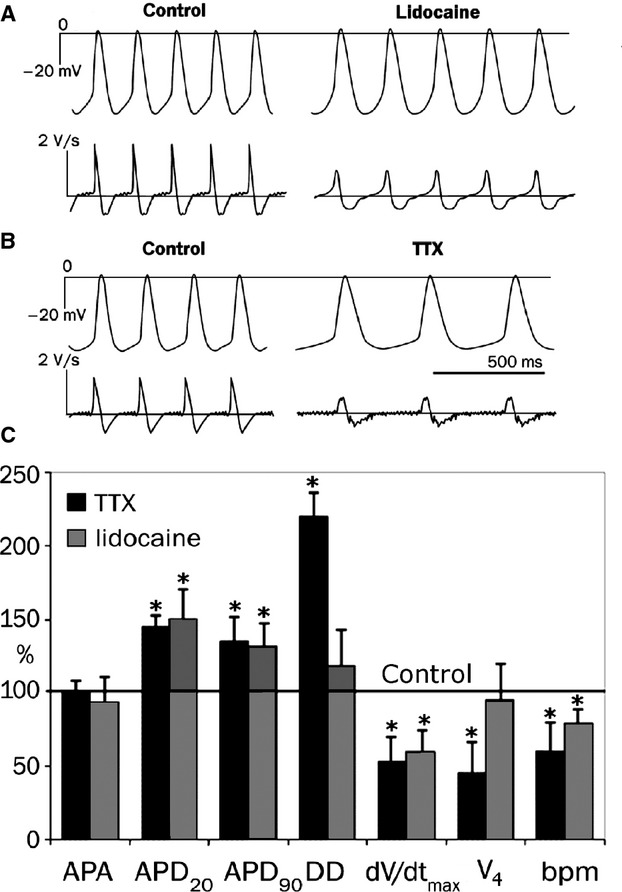
Effects of Na^+^-channel blockers (25 *μ*mol/L) on the generation of the AP of true pacemaker cells of mouse SAN. (A and B) the effects of TTX (25 *μ*mol/L) and lidocaine (25 *μ*mol/L) on the configuration of the AP and upstroke velocity. (C) the change in electrophysiological parameters of AP cells when added to saline solution with TTX and lidocaine (25 *μ*mol/L). Control is taken as 100%. Abbreviations: APA, action potential amplitude; APD_20_, APD_90_, duration of action potential at the level of 20% and 90%; DD, diastolic depolarization; d*V*/d*t*_max_, maximal upstroke velocity; V_4_, diastolic depolarization rate; bpm, beats per min. Values are means ± SEM or medians. *P < 0.05 versus control solution.

Lidocaine is a specific blocker of voltage-regulated Na^+^ channels. The substance is widely used in applied medicine as a local anesthetic. We are unaware of any data on lidocaine blocking of the L-type Ca^2+^ current. In the next set of experiments, we studied the effects of lidocaine at a concentration range of 5–500 *μ*mol/L on AP electrophysiological characteristics. Lidocaine considerably affected the AP duration at the 20% repolarization level, decreased the AP upstroke velocity (phase 0), and decreased the diastolic depolarization rate, V_4_, of strips spontaneously beating with a frequency of 315 ± 36 bpm (Fig.4A).

Tetrodotoxin is the most common blocker used in the analysis of electrical activity of voltage-gated Na^+^ channels in cardiomyocytes. We studied its effects on AP configuration of the slowest pacemaker cells in the mouse SAN.

After exposing TTX (25 *μ*mol/L) to a salt solution, we registered a d*V*/d*t*_max_ decrease from 1.8 to 1 V/sec (*n* = 3; *P* ≤ 0.05). The diastolic depolarization rate reduced by 49%. Therefore, the AP generation frequency decreased by 25–30% compared to the control (Fig.4B). It is notable that TTX exposure resulted in an increase in AP duration at the 20% repolarization level from 51 ± 6 to 65 ± 8 msec (*n*_strips_ = 3, *P* < 0.05). Consequently, AP duration increased at the 90% repolarization level. These data provide evidence that during TTX d*V*/d*t*_max_ inhibition, the AP duration increases at the 20% repolarization level, and the DD rate decreases by almost twice that of the control.

## Discussion

On the basis of the data obtained using the microelectrode technique, we found the location of true and latent pacemaker cells in the bifurcation of SAN artery area (Fig.1). The APs configurations from the SAN artery area were ultimately categorized into one of three main types: the true, the latent and the auricular like APs. For the first time a few APs in cells (*n* = 7) of the right SA ring bundle were measured (Table1). The dose-dependent effects of 4AP in concentrations from 0.1 to 5.0 mmol/L on the APs parameters were studied.

The dose-dependent effects of 4AP (0.1–5.0 mmol/L) on the electrophysiological parameters were studied. It was found that 4AP in concentration more than 0.5 mmol/L lengthened the APD_20_ in all types of cells. The analysis of curves of APD_20_ depending on 4AP concentration clearly demonstrated that the auricle type cells were the most sensitive (EC_50_ – 0.1 mmol/L), whereas the cells with the slowest d*V*/d*t*_max_ were the least sensitive (EC_50_ – 0.6 mmol/L).

It should be mentioned that the blocker 4-AP in concentration 0.1 mmol/L extended APD_20_ in atrial cells and did not affect the APs formation in the pacemaker cells. Therefore, the ultrafast potassium current *I*_Kur_ participates in the formation of APs in atrial cells and is inactivated in sinoauricular node cells of the mouse.

At the same time, the 4AP in millimolar concentrations (0.5–5 mmol/L) did not affect the duration and velocity of final repolarization phase in the pacemaker cells. Consequently, the *I*_Kr_ current was not inhibited by 4AP in this concentration. In cells of auricle and ventricle of rabbit and human hearts, the same concentration of 4AP affected the final repolarization phase (Yue et al. 1996; Mitcheson and Hancox 1999).

We, as the other authors (Lei et al. 2000; Aréchiga-Figueroa et al. 2010), registered the decrease of d*V*/d*t*_max_ in all types of cells in mouse SA area under exposure of 4AP. The effect was stronger in auricle cells with high initial value of upstroke velocity (Fig.3). Therefore, we suppose that 4AP can reduce inward Na^+^ current indirectly.

To test this assumption, we used specific blockers of Na^+^ current. Indeed, we found that the lidocaine and TTX slowed d*V*/d*t*_max_ two times and lengthened APD_20_.

In several laboratories, at least four outward potassium currents in mammalian auricular cells have been described: ultrarapidly activated delayed rectifier current (*I*_Kur_), the transient outward current (*I*_to_), the rapidly activating component of delayed K^+^ current (*I*_Kr_), and the slowly activating current (*I*_Ks_) (Yue et al. 1996; Boyett et al. 1998; Mitcheson and Hancox 1999; Nerbonne and Kaas 2005). Outward voltage-dependent K^+^ currents play a pivotal role in the repolarization of pacemaker cells and a working myocardium. Currently, more than 11 subfamilies of potential-dependent K^+^ channels have been identified. Among all of the channels in the mammalian heart, the K_v_1.2, K_v_1.4, K_v_1.5, K_v_ 2.1, and K_v_ 4.2 channels are characterized by a high template-RNA expression level (Nerbonne and Kaas 2005; Kodirov et al. 2010).

The detection of a 4AP-sensitive current in the SAN cells of mammals was previously performed on rabbits (Uese et al. 1999; Lei et al. 2000). Early work on the biophysical properties of rapid short-time current (*I*_to_) demonstrated that the current was suppressed by 4AP (0.3 mmol/L) at 50%, whereas current intensity was decreased by two-fold compared to auricular cells (Nerbonne and Kaas 2005). The 4AP (0.5 mmol/L) exposure prolonged the AP duration (Uese et al. 1999; Lei et al. 2000). Discussion of the optimal identification of K^+^ currents in cardiomyocytes continues (Boyett et al. 2001; Verkerk and van Ginneken 2001; Cha et al. 2009; Kharche et al. 2011). However, K^+^ currents are difficult to separate using different holding potentials. This problem is rarely solved or is solved by identification of K^+^ currents with pharmacological drugs (Kodirov et al. 2010).

The mechanism of blocking the K_v_4.2, K_v_4.3 channels by 4AP differ from the blocking mechanism of K_v_1.4 channels in ventricle cells of rat, rabbit and human 4AP in concentration of 0.14, 0.33, and 0.67 mmol/L correspondingly blocked *I*_to_ with IC_50_ (Ridley et al. 2003).

The structure of *I*_to_ channels is still debated (Nerbonne and Kaas 2005; Kodirov et al. 2010). On the basis of the detailed AP studies of pacemaker cells, for the first time, we have shown that 4AP blockers significantly prolonged the “plateau” phase but not the final repolarization phase in mouse SAN cells. This suggests that the current *I*_to_ makes a significant contribution to the generation of phase of repolarization. Evidently, in pacemaker cells a current flow through the channels formed by the K_v_ 4.2 or K_v_ 4.3 *α*-subunits (Ridley et al. 2003).

The data in this study do not allow the explanation of the upstroke velocity AP decrease of SAN cells. This phenomenon was described in this study for the first time and proceeds in the presence of 4AP. However, there are several hypotheses about phase 0 translation into phase 2 under 4AP exposure: (1) transient outward K^+^ current (*I*_to_) is in the final rapid depolarization phase, not at the early AP “plateau” phase; (2) blockage of the *I*_to_ current increases intracellular [K^+^]_i_ and, therefore, decreases conductivity of Na^+^ channels; and (3) an increase in the [K^+^]_i_ concentration slows the flow of an inward Ca^2+^ current (*I*_CaL_). The third hypothesis is, in our opinion, not likely because *I*_CaL_ current inhibition with a specific blocker (i.e., nifedipine) is known to result in a shortening of the AP duration (Kodama et al. 1997). We have shown that lidocaine and TTX exposition increased duration of action potential at the level of 20% (Fig.4C). Consequently, an APD_20_ increase is thought to depend on the inhibition of conductivity by voltage-gated inward Na^+^ channels.

Thus, for the first time, it has been demonstrated that a 4AP (0.5 mmol/L) blocker lengthened the duration of the AP “plateau” phase in pacemaker (d*V*/d*t*_max_ ≈ 3 V/sec) and auricular cells of the mouse SA heart area. This increase in duration was accompanied by the decrease in an upstroke velocity AP. The use of drugs on basis of pyridines, which can specifically regulate the AP duration time of SAN cells to increase the AP “plateau” phase duration, is a promising treatment direction.

The transient 4AP sensitive outward K^+^ current, *I*_to_, is one of the ionic membrane currents involved in the repolarization of SAN APs. This current was found in rabbit (Uese et al. 1999; Lei et al. 2000).

The presence of sensitive to 4AP current in the mouse SAN was demonstrated by us. The blocking of Ito prolonged APD_20_ and reduced d*V*/d*t*_max_. As a result, the spike of AP lengthened at 15–25%. The physiological role of *I*_to_ current is still unclear. We suggest that it can control the value of *I*_Na_ current during the APD_20._ The increase of APD_20_ and AP spike can result in arrhythmia (Zhao et al. 2012).

Now, we have a better understanding of cellular electrophysiology in normal cells of SA area mouse. But, much remains to be accomplished and this will be done through continued collaboration of basic and clinical scientists based on the foundations laid by studies pharmacology of cardiac K^+^- channels.
